# Significant association between systemic immune-inflammation index and stroke

**DOI:** 10.1097/MD.0000000000042979

**Published:** 2025-07-18

**Authors:** Dongfeng Wang, Lei Ma, Zhenqiang Li, Gengfan Ye, Maosong Chen

**Affiliations:** a Department of Neurosurgery, Ningbo Medical Center Lihuili Hospital, Ningbo, People’s Republic of China.

**Keywords:** cross-sectional, general population, NHANES, stroke, systematic immune-inflammation index

## Abstract

The study aimed to investigate the relationship between the systematic immune-inflammation index (SII) and stroke among American adults using data from the National Health and Nutrition Examination Survey from 2011 to 2020. The SII was calculated as the platelet count multiplied by the neutrophil count divided by the lymphocyte count. Weighted multivariate logistic analysis was used to estimate the relationship between SII and stroke, with subgroup and sensitivity analyses conducted to explore potential moderators and ensure result stability. The findings revealed that a higher SII was associated with increased susceptibility to stroke, particularly in females, after adjusting for covariates. Further prospective studies are needed to investigate the relationship between SII and different subtypes of stroke.

## 1. Introduction

Stroke, comprising ischemic and hemorrhagic subtypes, ranks as the second-leading cause of death and disability-adjusted life years worldwide.^[1]^ In the United States in 2018, stroke stood as the fifth-leading cause of death, representing 5.2% of all deaths, a 1.0 percentage point increase from 2017.^[2]^ Despite advancements in understanding stroke pathophysiology and the availability of therapies, the global burden of stroke-related morbidity and mortality remains significant. Therefore, there is a critical need for methods to assess stroke risk and severity, as well as to identify modifiable risk factors to reduce the incidence and mitigate the impact of stroke. Cells involved in innate and adaptive immune responses play a crucial role in stroke outcomes. Neutrophils, for instance, play a detrimental role by increasing infarct volume, stroke severity, and hemorrhagic complications in the early stages of stroke.^[3]^ They contribute to blood-brain barrier disruption through the release of matrix metalloproteinases, such as MMP-9, which promote thrombosis and inflammation, leading to cerebral edema.^[4–6]^ Neutrophils also interact with platelets, triggering the release of inflammatory mediators.^[7]^ Furthermore, stroke can impact intestinal barrier function through the microbiota-gut-brain axis, exacerbating systemic inflammation.^[8]^ Additionally, splenic macrophages can release inflammatory cytokines, further amplifying systemic inflammatory responses following brain injury.^[9]^

The systemic immune-inflammation index (SII), calculated based on lymphocyte, neutrophil, and platelet counts, is a novel indicator reflecting the systemic inflammatory and immune status in humans.^[10–12]^ Previous studies have shown its predictive value in long-term survival for cancer patients and elderly individuals with non-ST-elevation myocardial infarction.^[12–14]^ Xiao et al established a linear relationship between SII levels and mortality in patients with congestive heart failure and stroke.^[15]^ Meta-analyses have further demonstrated that higher SII values are associated with poorer clinical outcomes and increased incidence of hemorrhagic transformation in stroke patients.^[16]^ Additionally, the Dongfeng–Tongji cohort study revealed a significant link between elevated SII values and ischemic stroke risk. Despite these findings, the relationship between SII and stroke in the adult US population remains unclear. This study aims to investigate this association and explore potential moderators affecting this relationship, hypothesizing that higher SII values may indicate increased susceptibility to stroke.

## 2. Methods

### 2.1. Study population

**Figure 1.**
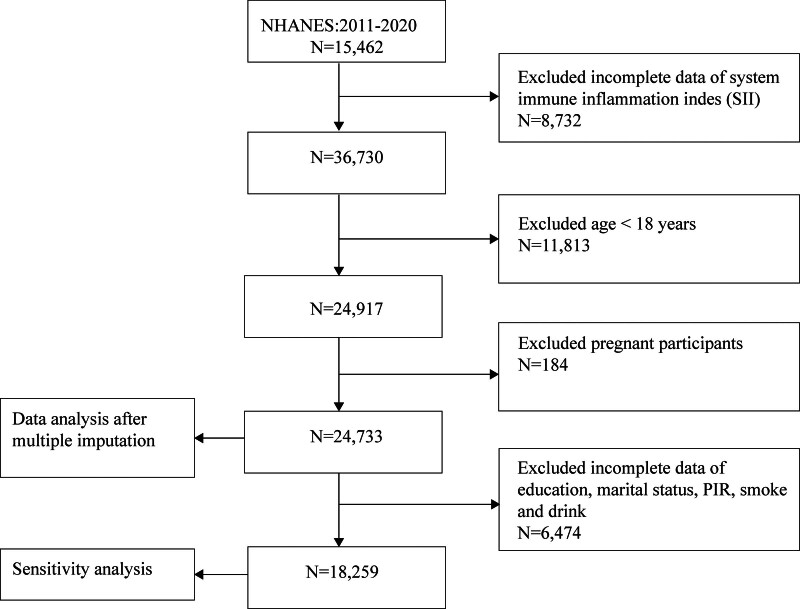
Flowchart of participant selection from the National Health and Nutrition Examination Survey 2011 to 2020.

Data from the National Health and Nutrition Examination Survey (NHANES) website were utilized for this study. NHANES is a comprehensive cross-sectional study conducted by the National Center for Health Statistics to evaluate the health and nutrition status of the noninstitutionalized civilian population in the US. The study employs a complex, multistage probability sampling design and is conducted biennially. Approval for the research was granted by the National Center for Health Statistics Research Ethics Review Board under the protocol number [Continuation of Protocol #2018-01], ensuring that all participants provided informed consent. As shown in Figure 1, a total of 45,462 participants from 5 NHANES cycles (2011–2020) were included in the analysis. Participants with missing SII data (N = 8732), those under 18 years of age (N = 11,813), and pregnant women (N = 184) were excluded based on the predetermined criteria. Multiple imputation was then used to address missing data for eligible participants.

### 2.2. The calculation of SII

Lymphocyte, neutrophil, and platelet counts were measured through comprehensive blood counts on samples with a concentration of 1000 cells/μL using an automated hematology analyzer (DxH-800 Analyzer, Beckman Coulter, Brea). The detailed methodology of the comprehensive blood count and other laboratory tests can be found on the NHANES website. The SII was calculated using the following equation^[17]^:


$$\begin{array}{l} \text{S}II=(\;platelet\;\;\;count\times neutrophil\;\;\;count)/(lymphocyte\;\;\;count) \end{array}$$

### 2.3. Definition of stroke

The NHANES data includes participants’ interview responses used to assess their medical history. Participants who reported being told by a doctor or health professional that they had experienced a stroke were classified as having had a stroke.

### 2.4. Covariates

This study investigated potential covariates influencing the association between the SII and stroke. Demographic factors such as sex, age, ethnicity, education level, marital status, poverty-to-income ratio (PIR), smoking and alcohol consumption were considered. Physiological parameters like metabolic equivalent (MET), diastolic blood pressure, systolic blood pressure (SBP), waist circumference, and body mass index (BMI) were also analyzed. Additionally, biochemical parameters including creatinine, serum uric acid, aspartate aminotransferase, alanine transaminase, blood urea nitrogen, glutamic acid (GLU), glycine, triglyceride, cholesterol (TC), low-density lipoprotein, and high-density lipoprotein concentrations were examined. The study also looked into health conditions such as hyperlipidemia, hypertension, diabetes, congestive heart failure, and coronary heart disease to determine their impact on stroke risk.

### 2.5. Statistical analysis

The NHANES utilized a complex sampling design, incorporating sample clusters, strata, and weights to analyze the data. Following NHANES guidelines, the sample weight used in the study was one fifth of the 2-Year Mobile Examination Center examination weight, combining data from 5 NHANES research cycles. Baseline characteristics were presented as weighted means and standard deviations for continuous variables, and weighted percentages for categorical variables. Statistical comparisons between non-stroke and stroke groups were conducted using weighted chi-square tests or weighted Student’s *t* tests. To address missing data for certain covariates such as level of education, marital status, PIR, and smoking and alcohol-drinking status, a “multiple imputations then deletion” method was employed to enhance the statistical power of the study. Multiple imputations by chained equations were used due to the expected missing data resulting from the complex NHANES design. Weighted multivariate logistic regressions were carried out to assess the relationship between the SII and stroke, controlling for various confounding variables. Model 1 adjusted for sex and age, Model 2 included ethnicity, level of education, marital status, PIR, smoking and alcohol-drinking status in addition to sex and age, while Model 3 further controlled for risk factors for stroke such as hyperlipidemia, hypertension, diabetes, coronary heart disease, and chronic heart failure. Data extraction and analysis were performed using the “nhanesR” package.

To investigate the potential factors influencing the relationship between the SII and stroke, participants were categorized into subgroups based on age (<60 or ≥60 years), sex (female or male), ethnicity (non-Hispanic black, non-Hispanic white, or other ethnicity), education level (above high school, high school, or below high school), smoking history (no or yes to having smoked at least 100 cigarettes), alcohol consumption (no or yes), presence of hypertension (no or yes), hyperlipidemia (no or yes), and diabetes (no or yes). An interaction analysis was also conducted to assess the variability in these relationships among the subgroups. Sensitivity analysis was carried out by excluding participants with incomplete data on education level, marital status, PIR, smoking, and alcohol use. Weighted multivariate logistic regression models were then applied to the remaining participants to explore the association between the SII and stroke. Additionally, the potential impact of confounding variables such as MET, SBP, GLU concentration, and TC concentration was assessed using the most controlled models. All statistical analyses were performed using R software (version 4.2.1; https://www.R-project.org), with a significance level set at *P* < .05.

## 3. Results

### 3.1. Baseline characteristics

The final sample included 24,733 participants with an average age of 48.5 years, consisting of 48.8% men and 51.2% women. A total of 998 participants (4.04%) had experienced a stroke, and this group exhibited significantly higher SII values compared to those who had not had a stroke. Significant differences between the 2 groups were observed in various factors such as age, marital status, education level, ethnicity, PIR, alcohol and smoking habits, MET, BMI, waist circumference, as well as various blood parameters and clinical conditions associated with stroke risk. Detailed clinical and biochemical characteristics of participants with and without a history of stroke are presented in Table 1.

**Table 1:** Baseline characteristics.

	Total	Non-stroke group	Stroke group	*P* value
Number of participants	24,733 (100.0)	23,735 (100.0)	998 (100.0)	
Age (year)	48.5 ± 18.4	47.7 ± 18.3	65.8 ± 12.8	<.001
Sex (%)				.883
Male	12,076 (48.8)	11,591 (48.8)	485 (48.6)	
Female	12,657 (51.2)	12,144 (51.2)	513 (51.4)	
Ethnicity (%)				<.001
Non-Hispanic White	9048 (36.6)	8612 (36.3)	436 (43.7)	
Non-Hispanic Black	5766 (23.3)	5461 (23.0)	305 (30.6)	
Other Race	9919 (40.1)	9662 (40.7)	257 (25.8)	
Education (%)				.015
Below high school level	2671 (10.8)	2514 (10.6)	157 (15.7)	
High school	15,379 (62.2)	14,887 (62.7)	492 (49.3)	
Above high school	6683 (27.0)	6334 (26.7)	349 (35.0)	
Marital status				.004
Married/Living with partner	16,452 (66.5)	15,883 (66.9)	569 (57.0)	
Widowed/Divorced/Separated	4169 (16.9)	3815 (16.1)	354 (35.5)	
Never married	4112 (16.6)	4037 (17.0)	75 (7.5)	
Poverty income ratio (PIR)	2.5 ± 1.6	2.5 ± 1.6	2.0 ± 1.4	<.001
Smoked at least 100 cigarettes in life (%)				<.001
No	14,532 (58.8)	14,131 (59.5)	401 (40.2)	
Yes	10,201 (41.2)	9604 (40.5)	597 (59.8)	
Drink (%)				.004
Never	6177 (25.0)	5938 (25.0)	239 (23.9)	
Former	1623 (6.6)	1484 (6.3)	139 (13.9)	
Current	16,933 (68.5)	16,313 (68.7)	620 (62.1)	
Metabolic equivalent minutes (MET)	1129.9 ± 1377.5	1141.0 ± 1380.8	866.4 ± 1268.7	<.001
BMI (kg/m^2^)	29.3 ± 7.3	29.3 ± 7.3	29.9 ± 7.1	.012
SBP (mm Hg)	123.8 ± 18.4	123.4 ± 18.1	133.7 ± 21.7	<.001
DBP (mm Hg)	71.7 ± 12.8	71.7 ± 12.7	71.3 ± 15.0	.269
Platelet count	240.3 ± 62.8	240.6 ± 62.3	233.4 ± 71.5	<.001
Neutrophils count	4.2 ± 1.7	4.2 ± 1.7	4.5 ± 1.8	<.001
Lymphocyte count	2.2 ± 2.6	2.2 ± 2.6	2.0 ± 1.3	.049
SII	500.2 ± 331.5	495.6 ± 303.8	545.2 ± 341.8	<.001
Creatinine (mg/dL)	11233.7 ± 7424.6	11253.9 ± 7434.2	10723.7 ± 7163.3	.033
SUA	322.4 ± 85.8	321.7 ± 85.4	340.3 ± 94.2	<.001
BUN (mmol/L)	5.0 ± 2.2	5.0 ± 2.1	6.3 ± 3.3	<.001
ALT (IU/L)	24.0 ± 20.5	24.1 ± 20.7	21.0 ± 14.3	<.001
AST (IU/L)	24.4 ± 16.8	24.4 ± 16.9	23.5 ± 12.1	.116
GLU (mmol/L)	5.7 ± 2.2	5.7 ± 2.2	6.5 ± 2.9	<.001
GLY (mmol/L)	5.8 ± 1.1	5.8 ± 1.1	6.2 ± 1.3	<.001
HDL (mmol/L)	1.4 ± 0.4	1.4 ± 0.4	1.4 ± 0.4	.506
LDL (mmol/L)	2.8 ± 0.9	2.8 ± 0.9	2.6 ± 1.0	<.001
TC (mmol/L)	4.8 ± 1.1	4.9 ± 1.1	4.6 ± 1.1	<.001
TG (mmol/L)	1.6 ± 1.3	1.6 ± 1.4	1.7 ± 1.1	.763
Hypertension				<.001
No	15,838 (64.0)	15,595 (65.7)	243 (24.3)	
Yes	8895 (36.0)	8140 (34.3)	755 (75.7)	
Hyperlipidemia				<.001
No	16,424 (66.4)	16,014 (67.5)	410 (41.1)	
Yes	8309 (33.6)	7721 (32.5)	588 (58.9)	
Diabetes				<.001
No	21,354 (86.3)	20,684 (87.1)	670 (67.1)	
Yes	3379 (13.7)	3051 (12.9)	328 (32.9)	
Coronary heart disease				<.001
No	23,770 (96.1)	22,955 (96.7)	815 (81.7)	
Yes	963 (3.9)	780 (3.3)	183 (18.3)	
Congestive heart failure heart failure				<.001
No	23,904 (96.6)	23,081 (97.2)	823 (82.5)	
Yes	829 (3.4)	654 (2.8)	175 (17.5)	

ALT = alanine transaminase, AST = aspartate aminotransferase, BMI = body mass index, BUN = blood urea nitrogen, DBP = diastolic blood pressure, GLU = glutamic acid, GLY = glycine, HDL = high-density lipoprotein, LDL = low-density lipoprotein, MET = metabolic equivalent, PIR = ratio of family income to poverty, SBP = systolic blood pressure, SII = systematic immune-inflammation, SUA = serum uric acid, TC = total cholesterol, TG = triglycerides.

### 3.2. Association between SII values and stroke

The relationship between SII values and stroke risk was analyzed in different models (Table 2). In Model 1, adjusting for age and sex, an increase in SII value was associated with a higher risk of stroke in subgroups based on continuous data (modified odds ratio [OR] = 1.11, 95% CI = 1.07–1.15) and categorical data (OR [quartile 4 vs quartile 1] = 1.69, 95% CI = 1.40–2.03, P for trend < 0.001). Model 2 further controlled for ethnicity, level of education, marital status, PIR, smoking, and alcohol-drinking status, showing similar relationships for continuous data (OR = 1.10, 95% CI = 1.06–1.14) and categorical data (OR [quartile 4 vs quartile 1] = 1.74, 95% CI = 1.43–2.11, *P* for trend < .001). In the fully adjusted Model 3, after accounting for additional risk factors for stroke (hypertension, chronic heart failure, coronary heart disease, diabetes, and hyperlipidemia), the relationships remained significant with an OR (95% CI) of 1.06 (1.02–1.10) for continuous data and 1.45 (1.19–1.77) for categorical data.

**Table 2:** Association between systemic immune-inflammation index and stroke.

	Continuous SII			Categorical SII					
	OR (95% CI)	β	*P* for β	Quartile 1 OR (95% CI)	Quartile 2 OR (95% CI)	Quartile 3 OR (95% CI)	Quartile 4 OR (95% CI)	OR_std	*P* for trend
SII value				2.5 (0.1, 3.0)	3.6 (3.1, 4.0)	4.5 (4.1, 5.1)	6.1 (5.1, 35.2)		
Number of cases/participants	998/23,735			199/6222	243/6786	239/5834	317/5891		
Model 1*	1.11 (1.07–1.15)	0.102	<.001	Ref.	1.02 (0.84–1.24)	1.18 (0.97–1.43)	1.69 (1.40–2.03)	1.19 (1.12–1.26)	<.001
Model 2†	1.10 (1.06–1.14)	0.096	<.001	Ref.	1.13 (0.92–1.37)	1.28 (1.04–1.56)	1.74 (1.43–2.11)	1.18 (1.11–1.25)	<.001
Model 3‡	1.06 (1.02–1.10)	0.060	.001	Ref.	1.06 (0.87–1.30)	1.13 (0.92–1.39)	1.45 (1.19–1.77)	1.11 (1.04–1.18)	<.001

CI = confidence interval, OR = odds ratio, SII = systemic immune-inflammation index.

* Model 1 adjusted for age and sex.

† Model 2 adjusted for age, sex, ethnicity, education level, marital status, ratio of family income to poverty (PIR), and smoking and drinking status.

‡ Model 3 adjusted for age, sex, ethnicity, education level, marital status, PIR, smoking status, drinking status, hypertension, hyperlipidemia, diabetes, coronary heart disease, and chronic heart failure.

### 3.3. Subgroup analysis

The subgroup analysis, as presented in Table 3, demonstrated that the association between the SII and stroke was influenced by the characteristics of the participants. Specifically, a higher SII value was significantly linked to a greater susceptibility to stroke in participants aged 60 years or older, female participants, individuals of ethnicities other than non-Hispanic black or non-Hispanic white, those with a high-school education or lower, and participants without diabetes. Additionally, a stronger correlation between SII values and stroke was noted in participants who were nondrinkers compared to those who consumed alcohol, and in individuals with a normal BMI (<25 kg/m^2^) compared to those with obesity. However, the interaction analysis did not reveal significant effects of the aforementioned factors on the relationship between the SII and stroke, as indicated by the *P* values for the interactions ranging from .054 to .664.

**Table 3:** Subgroup analysis was conducted to examine the correlation between the systemic immune-inflammation index and stroke.

	Quartile 1	Quartile 2 OR (95% CI)	Quartile 3 OR (95% CI)	Quartile 4 OR (95% CI)	*P* for interaction
Age, years					.513
<60	Ref.	2.36 (0.66–8.39)	1.08 (0.25–4.63)	2.05 (0.51–8.32)	
≥60	Ref.	1.04 (0.85–1.27)	1.13 (0.91–1.40)	1.43 (1.17–1.76)	
Sex					.054
Male	Ref.	0.81 (0.61–1.08)	0.96 (0.71–1.30)	1.39 (1.04–1.84)	
Female	Ref.	1.34 (1.01–1.78)	1.30 (0.97–1.74)	1.47 (1.11–1.96)	
Ethnicity					.217
Non-Hispanic White	Ref.	1.03 (0.73–1.46)	0.90 (0.64–1.28)	1.31 (0.94–1.83)	
Non-Hispanic Black	Ref.	0.98 (0.71–1.35)	1.41 (1.01–1.99)	1.21 (0.85–1.72)	
Other Race	Ref.	1.25 (0.82–1.92)	1.37 (0.89–2.11)	1.89 (1.25–2.85)	
Education					.653
Below high school level	Ref.	1.51 (0.86–2.65)	1.64 (0.92–2.93)	2.04 (1.14–3.66)	
High school	Ref.	1.06 (0.80–1.42)	1.12 (0.83–1.50)	1.53 (1.16–2.01)	
Above high school	Ref.	0.92 (0.66–1.29)	1.00 (0.70–1.43)	1.17 (0.83–1.64)	
Smoked at least 100 cigarettes in life					.450
No	Ref.	1.04 (0.77–1.41)	0.99 (0.71–1.38)	1.53 (1.13–2.09)	
Yes	Ref.	1.05 (0.80–1.38)	1.22 (0.93–1.61)	1.41 (1.08–1.83)	
Drink					.445
No	Ref.	1.29 (0.92–1.79)	1.38 (0.98–1.95)	1.59 (1.13–2.23)	
Yes	Ref.	0.94 (0.73–1.22)	1.00 (0.77–1.30)	1.36 (1.06–1.75)	
BMI					.206
No	Ref.	0.99 (0.66–1.47)	1.50 (1.01–2.22)	1.71 (1.16–2.53)	
Yes	Ref.	1.09 (0.86–1.38)	1.04 (0.81–1.33)	1.38 (1.09–1.75)	
Hypertension					.664
No	Ref.	1.18 (0.80–1.74)	1.27 (0.84–1.91)	1.56 (1.04–2.32)	
Yes	Ref.	1.01 (0.80–1.28)	1.07 (0.84–1.37)	1.39 (1.10–1.76)	
Hyperlipidemia					.614
No	Ref.	1.11 (0.82–1.51)	1.00 (0.73–1.39)	1.45 (1.07–1.97)	
Yes	Ref.	1.02 (0.78–1.34)	1.22 (0.92–1.60)	1.45 (1.11–1.89)	
Diabetes					.397
No	Ref.	1.11 (0.88–1.41)	1.29 (1.01–1.65)	1.55 (1.22–1.98)	
Yes	Ref.	0.93 (0.63–1.36)	0.82 (0.56–1.21)	1.17 (0.81–1.69)	

BMI = body mass index, CI = confidence interval, OR = odds ratio.

### 3.4. Sensitivity analysis

In our sensitivity analysis of the relationship between the SII and stroke, we focused on participants with complete data by excluding those with incomplete data for education level, marital status, PIR, smoking, and alcohol consumption. We conducted weighted multivariate logistic regressions with various controls to examine the relationship between the SII and stroke. Additionally, we investigated the influence of confounding factors such as MET, GLU concentration, SBP, and TC concentration on this relationship using a comprehensive model. After excluding participants with incomplete data based on the primary study’s method, we analyzed 18,259 individuals with complete data. Our findings revealed that a higher SII value was associated with an increased risk of stroke, with categorical ORs (95% confidence intervals) of 1.66 (1.33–2.06) for Model 1, 1.16 (1.08–1.25) for Model 2, and 1.08 (1.01–1.17) for Model 3. The positive association between the SII and stroke persisted even after considering confounders such as MET, GLU concentration, SBP, and TC concentration in Model 4 and Model 5. More details on the sensitivity analysis can be found in Table 4.

**Table 4:** Sensitivity analysis was conducted to explore the relationship between the systemic immune-inflammation index and stroke occurrence.

	Continuous SII			Categorical SII					
	OR (95% CI)	β	*P* for β	Quartile 1 OR (95% CI)	Quartile 2 OR (95% CI)	Quartile 3 OR (95% CI)	Quartile 4 OR (95% CI)	OR_std	*P* for trend
Complete cases									
SII value				2.5 (0.1, 3.0)	3.5 (3.1, 3.9)	4.5 (4.0, 5.1)	6.1 (5.1, 35.2)		
Number of cases/participants				145/4591	155/4585	197/4798	226/4285		
Model 1*	1.10 (1.06–1.15)	0.097	<.001	Ref.	0.98 (0.78–1.24)	1.19 (0.95–1.48)	1.66 (1.33–2.06)	1.18 (1.10–1.26)	<.001
Model 2†	1.09 (1.05–1.14)	0.088	<.001	Ref.	1.07 (0.84–1.36)	1.27 (1.01–1.61)	1.68 (1.33–2.11)	1.16 (1.08–1.25)	<.001
Model 3‡	1.05 (1.00–1.10)	0.048	.029	Ref.	1.00 (0.78–1.27)	1.12 (0.88–1.41)	1.37 (1.08–1.73)	1.08 (1.01–1.17)	.004
Confounding effects									
SII value				2.5 (0.1, 3.0)	3.6 (3.1, 4.0)	4.5 (4.1, 5.1)	6.1 (5.1, 35.2)		
Number of cases/participants				199/6222	243/6786	239/5834	317/5891		
Model 4§	1.06 (1.02–1.1)	0.060	.001	Ref.	1.06 (0.87–1.30)	1.13 (0.92–1.39)	1.45 (1.19–1.77)	1.11 (1.04–1.18)	<.001
Model 5∥	1.06 (1.02–1.1)	0.0588	.001	Ref.	1.06 (0.86–1.29)	1.12 (0.91–1.37)	1.44 (1.18–1.75)	1.11 (1.04–1.18)	<.001

CI = confidence interval, OR = odds ratio, SII = systematic immune-inflammation.

* Model 1 adjusted for age and sex.

† Model 2 adjusted for age, sex, ethnicity, education level, marital status, ratio of family income to poverty (PIR), and smoking and drinking status.

‡ Model 3 adjusted for age, sex, ethnicity, education level, marital status, PIR, smoking status, drinking status, hypertension, hyperlipidemia, diabetes, coronary heart disease, and chronic heart failure.

§ Model 4 adjusted for all covariates in Model 3, and further adjusted for metabolic equivalent (MET).

∥ Model 5 adjusted for all covariates in Model 3, and further adjusted for MET, glutamic acid (GLU), systolic blood pressure (SBP), and total cholesterol (TC).

## 4. Discussion

The study found a significant association between the SII and risk of stroke. Specifically, individuals in the fourth SII quartile had a higher risk of stroke compared to those in the first quartile. Subgroup analysis suggested that this relationship was more prominent in participants aged 60 years or older, women, individuals with a normal BMI (< 25 kg/m^2^), or those without diabetes. However, these results should be interpreted with caution and require validation in future research, especially considering that only the interaction between SII and sex showed a trend towards statistical significance. Additionally, sensitivity analysis indicated that the association between SII and stroke risk was not influenced by dataset or confounding variables.

There is a known correlation between individual leukocyte subtype counts and cardiovascular events.^[18]^ However, the association between comprehensive blood count indicators and stroke incidence remains unconfirmed. The SII offers advantages over single blood-cell counts as it is less influenced by factors like fluid status and dehydration.^[19]^ Xu et al conducted a prospective cohort study involving 13,929 middle-aged and elderly Chinese adults, investigating the link between SII and the occurrence of cardiovascular disease (CVD) and its subtypes.^[18]^ Their results align with our findings, showing that elevated SII values were associated with increased susceptibility to stroke (HR = 1.22, 95% CI = 1.07–1.41) and specific subtypes like ischemic stroke (HR = 1.23, 95% CI = 1.06–1.44), but not with CVD risk, coronary heart disease, or acute coronary syndrome. A recent meta-analysis of studies from China and Turkey further supported these findings, indicating that higher SII values were linked to elevated risk of stroke (HR = 1.31, 95% CI = 1.07–1.60), ischemic stroke (HR = 1.31, 95% CI = 1.06–1.63), and hemorrhagic stroke (HR = 1.22, 95% CI = 1.10–1.37).^[20]^ Our current study builds upon these outcomes, revealing a similar relationship between SII values and stroke risk in the US population, suggesting the potential utility of SII values in predicting stroke onset.

The Stroke-Induced Immunodepression Syndrome (SII) integrates information on neutrophils, platelets, and lymphocytes, potentially reflecting pathways related to stroke.^[20,21]^ Neutrophils are one of the initial immune cell types to reach the brain poststroke,^[22]^ interacting with the cerebral endothelium to release reactive oxygen species, proteases, and cytokines that contribute to blood-brain barrier disruption, cerebral edema, and brain injury.^[23–25]^ Platelets are also crucial in the interplay between inflammatory and thrombotic cascades triggered by stroke.^[26]^ Upon activation, platelets adhere to damaged endothelium, releasing substances that promote platelet aggregation and activate coagulation pathways. Neutrophils and platelets collaborate to activate neutrophil elastase, which enhances matrix metalloproteinase 9 production, further disrupting the blood-brain barrier and releasing damaging molecules.^[4]^ Lymphocytes, on the other hand, regulate inflammation, with certain subtypes demonstrating antiatherogenic effects that could be beneficial in stroke scenarios.^[19]^ Regulatory T cells, for example, release anti-inflammatory cytokines like interleukin 10 (IL-10) to protect against ischemic stroke,^[27]^ while IL-10 stimulates B cells that can reduce infarct volume.^[4]^ CD4+, CD8+, and gamma delta T cells exacerbate stroke injury through a secondary pathway that involves IL-17 and interferon gamma.^[28]^ In conclusion, while the relationship between stroke occurrence and the SII remains unclear, there may be potential for secondary stroke prevention by focusing on medical screening for subpopulations with elevated SII values.

Subgroup analyses indicated a marginally significant impact of sex on the association between the SII value and stroke. Women face a disproportionately higher risk of stroke-related mortality and disability,^[4]^ partly attributed to their longer lifespan compared to men. Variability in the strength of the links between stroke and female-specific risk factors was observed. Additionally, sex-specific poststroke inflammatory feedback pathways contribute to differences in recovery and infarct volume between genders. Disparities in innate immunity between sexes emerge early in the inflammatory process, with women showing higher levels of Toll-like receptor 7 and more mature neutrophils compared to men. The study findings suggest a stronger relationship between the SII and stroke in women than in men, hinting at a potential sex-specific pathway connecting immune-inflammatory responses with the SII. Further research is necessary to elucidate the role of chronic systemic immunity and inflammation in the pathogenesis of stroke in women.

In this study, the authors extensively investigated the influence of demographic parameters, physiological and biochemical characteristics, and stroke risk factors on the relationship between the SII and stroke. Surprisingly, interaction tests did not reveal any moderation effects of the demographic parameters and stroke risk factors on this relationship.^[29]^ Older adults typically experience chronic low-grade inflammation due to age-related tissue damage. Previous research by Xu et al demonstrated a significant association between the SII and CVD in individuals aged 65 years or older but not in those younger than 65 years. Similarly, our study found a positive association between the SII and stroke only in participants aged 60 years or older, possibly due to their higher vulnerability to strokes. Adhering to healthy behaviors and maintaining recommended physiological and biochemical values are crucial for cardiovascular health.^[30]^ Metabolic syndrome, linked to an inflammatory state and increased CVD risk, showed stronger associations with SII values in individuals with diabetes mellitus or hypertension according to Xu et al Nevertheless, our results suggest that the SII may not always be a direct risk factor for stroke, emphasizing the need for prospective studies to confirm our subgroup analyses.

While our study benefits from a large sample size and the use of multiple imputation techniques, it is important to acknowledge several limitations. The cross-sectional design of the NHANES prevents us from establishing a causal relationship between the SII and stroke, highlighting the need for future studies with larger samples. Additionally, our study’s definition of stroke, while relatively accurate, did not differentiate between stroke subtypes, impacting the clarity of our results. Furthermore, reliance on self-reported information for some covariates may have introduced recall bias, potentially affecting the accuracy of our findings. Despite these limitations, our study indicates a significant association between higher SII values and increased susceptibility to stroke, particularly among women. This underscores the potential of the SII as an inflammatory marker for identifying stroke risk in the US population and its potential utility in clinical practice. However, given the diversity of stroke subtypes, further prospective research is warranted to explore the relationship between the SII and stroke.

## Author contributions

**Conceptualization:** Dongfeng Wang.

**Data curation:** Lei Ma.

**Formal analysis:** Zhenqiang Li.

**Methodology:** Dongfeng Wang.

**Writing – original draft:** Dongfeng Wang, Lei Ma, Zhenqiang Li.

**Writing – review & editing:** Gengfan Ye, Maosong Chen.
